# Decoding the Intricate Landscape of Pancreatic Cancer: Insights into Tumor Biology, Microenvironment, and Therapeutic Interventions

**DOI:** 10.3390/cancers16132438

**Published:** 2024-07-02

**Authors:** Antonella Argentiero, Alessandro Andriano, Ingrid Catalina Caradonna, Giulia de Martino, Vanessa Desantis

**Affiliations:** 1 Istituto Tumori “Giovanni Paolo II”, 70124 Bari, Italy; 2 Department of Precision and Regenerative Medicine and Ionian Area, Pharmacology Section, Medical School, University of Bari Aldo Moro, 70124 Bari, Italy; a.andriano.aui@gmail.com (A.A.); ingrid.caradonna@uniba.it (I.C.C.); 3 Department of Biosciences, Biotechnology and Environment, University of Bari Aldo Moro, 70121 Bari, Italy; giulia.demartino@uniba.it

**Keywords:** pancreatic cancer, tumor microenvironment, cancer-associated fibroblasts, macrophages, tumor-associated macrophages, TGF-beta signaling, Notch signaling, PD-L1

## Abstract

**Simple Summary:**

Pancreatic ductal adenocarcinoma (PDAC) is an aggressive malignancy with a complex tumor microenvironment (TME) that promotes cancer progression. The tumor stroma is characterized by cancer-associated fibroblasts (CAFs) that secrete factors stimulating tumor growth, invasion, and immunosuppression. Macrophages tend to polarize toward a pro-tumor phenotype. Various other components, including endothelial cells, pericytes, and neural cells, likely contribute to the complexity of the PDAC TME. Epidemiological investigations have highlighted obesity as a risk factor for pancreatic cancer, suggesting that adipocytes may also play a significant role. This manuscript offers an overview of the main actors in TME, as well as dysregulated signaling pathways and gene mutations. It underlines how strategies modulating the TME hold promise for improving outcomes. Lastly, it describes how integrating multi-omic approaches may represent a future trajectory to explore novel therapeutic possibilities.

**Abstract:**

Pancreatic ductal adenocarcinoma (PDAC) presents significant oncological challenges due to its aggressive nature and poor prognosis. The tumor microenvironment (TME) plays a critical role in progression and treatment resistance. Non-neoplastic cells, such as cancer-associated fibroblasts (CAFs) and tumor-associated macrophages (TAMs), contribute to tumor growth, angiogenesis, and immune evasion. Although immune cells infiltrate TME, tumor cells evade immune responses by secreting chemokines and expressing immune checkpoint inhibitors (ICIs). Vascular components, like endothelial cells and pericytes, stimulate angiogenesis to support tumor growth, while adipocytes secrete factors that promote cell growth, invasion, and treatment resistance. Additionally, perineural invasion, a characteristic feature of PDAC, contributes to local recurrence and poor prognosis. Moreover, key signaling pathways including Kirsten rat sarcoma viral oncogene (KRAS), transforming growth factor beta (TGF-β), Notch, hypoxia-inducible factor (HIF), and Wnt/β-catenin drive tumor progression and resistance. Targeting the TME is crucial for developing effective therapies, including strategies like inhibiting CAFs, modulating immune response, disrupting angiogenesis, and blocking neural cell interactions. A recent multi-omic approach has identified signature genes associated with anoikis resistance, which could serve as prognostic biomarkers and targets for personalized therapy.

## 1. Introduction

Pancreatic ductal adenocarcinoma (PDAC) is a devastating malignancy with an average 5-year survival rate of less than 11% [1]. Conventional therapies are largely ineffective, with less than 18% of patients surviving beyond the first year [2]. It ranks as the fourth leading cause of cancer-related deaths in the United States, with an estimated 60.430 new cases and 48.220 deaths in 2021 [3]. The aggressive nature, rapid disease progression, and treatment resistance underscore the urgent need for a deeper understanding of its underlying biology to develop more effective therapeutic strategies.

This study offers an overview of the main actors in a tumor microenvironment (TME), as well as dysregulated signaling pathways and gene mutations. It underlines how strategies modulating the TME hold promise for improving outcomes and describes the main trends and future trajectories for novel therapeutic possibilities.

One of the hallmarks of PDAC is its complex and dynamic (TME), which plays a critical role in disease progression and treatment resistance. Within TME, fibroblasts respond to increased local cytokine levels by transitioning into cancer-associated fibroblasts (CAFs), which exhibit a pro-tumorigenic phenotype. CAFs are abundant within solid tumors as they can comprise up to 80% of the tumor mass [4]. CAF-mediated pro-tumorigenic effects involve various mechanisms, such as extracellular matrix (ECM) remodeling, direct cell–cell interactions, and the secretion of soluble factors. Peritumoral fibroblasts significantly influence tumor cells, promoting enhanced proliferation, migration, and invasion, partly through interleukin-6 (IL-6)-mediated signaling pathways. These complex interactions between CAFs and tumor cells play a crucial role in driving the initiation and progression of distant metastases [5].

Moreover, macrophages present in the tumor stroma, known as Tumor-Associated Macrophages (TAMs), represent one of the most abundant cell populations of the immune system. The traditional perspective distinguishes between two main types of macrophage polarization: M1 and M2; although TAMs show dynamic modifiability in the development of this tumor, they tend to polarize towards the M2 phenotype with pro-tumoral effects. Among these effects are the promotion of tumorigenesis, the induction of immunosuppression, and the production of growth factors, proteolytic enzymes, and various immune checkpoint receptors in T cells [6]. TAMs also play a critical role in the regulation of metastasis, secreting matrix proteins and proteases, including serine proteases, matrix metalloproteinases (MMPs), and cathepsins [7].

The vasculature of the TME, composed of endothelial cells and pericytes, plays a critical role in tumor angiogenesis. Endothelial cells secrete growth factors that stimulate angiogenesis and recruit pericytes to stabilize newly formed blood vessels. In turn, pericytes secrete cytokines and growth factors that modulate the microenvironment [8,9]. Neural cells, including neurons and glial cells, are emerging as important regulators of the PDAC TME. These neural components secrete neurotransmitters, neuropeptides, and neurotrophic factors that influence cancer cell proliferation, migration, and invasion [10]. Interactions among these components create a dynamic ecosystem that shapes the behavior of tumor cells. Cancer cells undergo genetic and epigenetic alterations that confer their survival advantage, proliferation, and invasive capabilities [11]. Additionally, PDAC cells themselves secrete neurotrophic factors that attract and activate neural cells, further promoting tumor growth and invasion [12]. Common genetic mutations in genes such as KRAS, tumor protein 53 (TP53), cyclin-dependent kinase inhibitor 2A (CDKN2A), and mothers against decapentaplegic homolog 4 (SMAD4) are frequently observed and are associated with poor prognosis [13,14]. Additionally, metabolic reprogramming, characterized by increased glycolysis and reduced mitochondrial respiration, contributes to the aggressive phenotype of neoplastic cells [15].

## 2. PDAC Tumor Microenvironment

**Figure 1:**
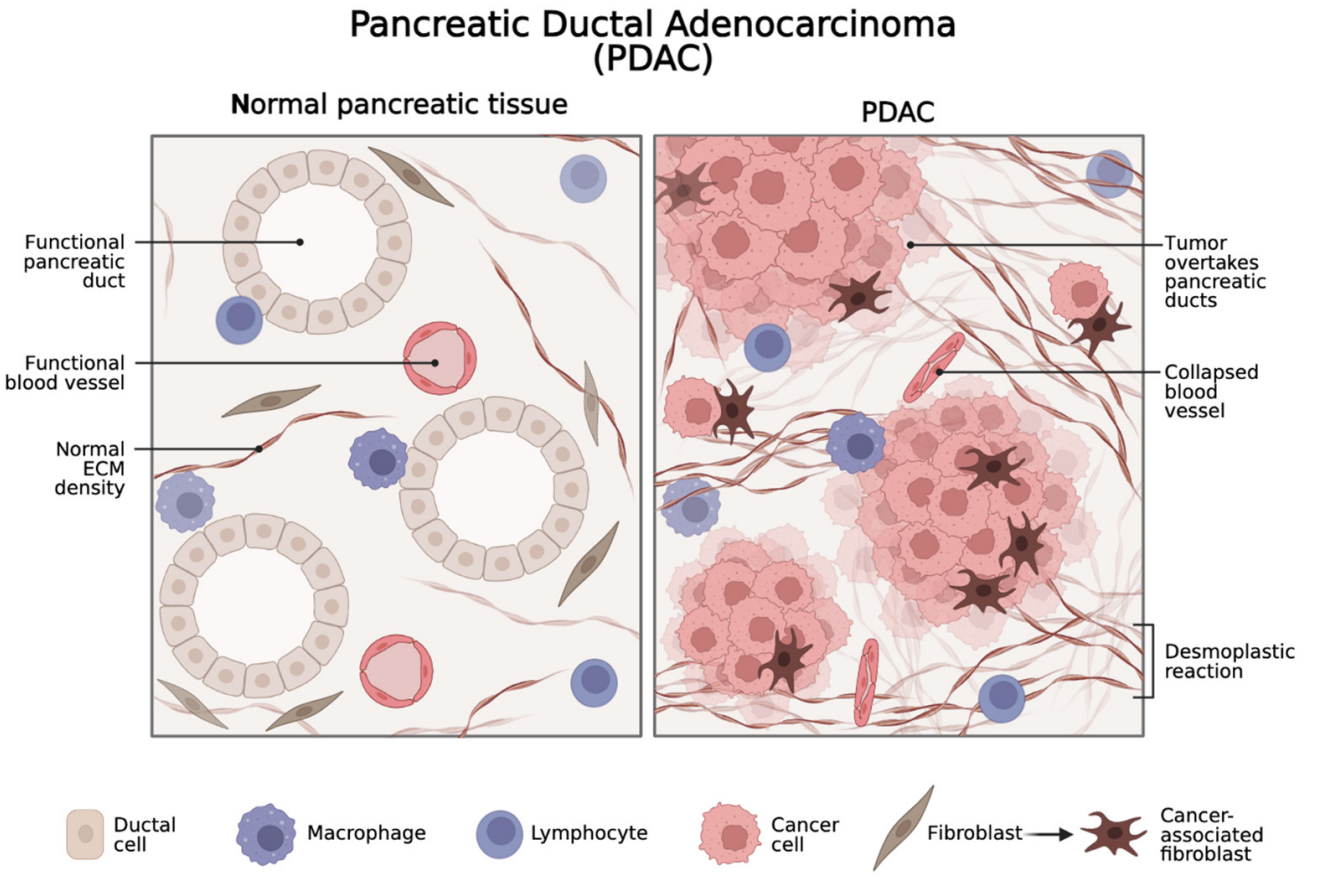
Schematic representation of the pancreatic ductal adenocarcinoma (PDAC) tumor microenvironment (TME). In normal pancreatic tissue, stromal cells such as fibroblasts play a supportive role in tissue homeostasis and repair. However, cancer-associated fibroblasts (CAFs) are activated and secrete a variety of factors that promote tumor growth and invasion. CAFs contribute to the dense desmoplastic stroma that is a hallmark of pancreatic cancer, which can limit the delivery of chemotherapy drugs to the tumor.

The immune system and its various components are intricately involved in the TME of PDAC. Immune cells, including T cells, B cells, natural killer (NK) cells, dendritic cells (DCs), and macrophages, infiltrate the extracellular matrix (ECM) and interact with cancer cells and other TME components. However, the immune response in PDAC is complex, characterized by a delicate balance between pro-tumor and anti-tumor signals. Oncogenic cells secrete chemokines that attract immune cells to the TME but also express immune checkpoint molecules that inhibit T-cell activation and function, resulting in immune evasion [16,17]. The interplay between these immune signals has a significant impact on disease progression and treatment outcomes. The TME encompasses a diverse range of cellular components, including cancer cells, CAFs, immune cells, endothelial cells, pericytes, and neural cells, along with the ECM [18] (Figure 1).

### 2.1. Cancer-Associated Fibroblasts (CAFs)

CAFs in PDAC exhibit intricate ontogeny and differentiation, driving tumor-associated desmoplasia. They actively remodel the ECM, thus facilitating cancer cell invasion and metastasis [19], enhancing the expression of MMPs that promote these processes, increasing tissue stiffness through matrix crosslinking and remodeling, which leads to hypoxia and a more aggressive cancer phenotype. Additionally, CAFs play a role in restricting drug delivery, triggering pro-survival signals in cancer cells, and supporting the establishment of tumors at secondary sites. They also aid in immune exclusion and provide survival cues within the TME [20].

They can arise from various cell types, including tissue-resident pancreatic stellate cells (PSCs), bone marrow-derived progenitors, and adipose tissue-derived mesenchymal stem cells. These cells can differentiate into different phenotypes with unique functions and signaling molecules [21]. For example, Dominguez et al. [22] identified two primary CAF lineages using single-cell RNA sequencing, unlike Elyada et al. [23], who revealed three distinct populations: TGFβ-driven myofibroblastic, IL-1α-driven inflammatory, and antigen-presenting expressing CD74 and MHC molecules. Notably, TGFβ-driven CAFs showed resistance to anti-PD-L1 immunotherapy.

Moreover, α-SMA and fibroblast activation protein (FAP) are primary markers for CAFs; however, deleting each subtype produces opposing effects. In vitro experiments showed that removing α-SMA^+^ cells during PDAC development results in poorly differentiated tumors and reduced survival. Conversely, eliminating FAP^+^ cells boosts cytotoxic CD8^+^ T-cell activity and slows pancreatic tumor growth [24]. The tumor necrosis factor (TNF) produced by neutrophils triggers an excessive production of C-X-C motif chemokine ligand 1 (CXCL1) in both tumor cells and cancer-associated fibroblasts (CAFs), resulting in the suppression of T cells. In response to environmental stressors (a.k.a. chemotherapy), CAFs and pancreatic cells release C-X-C motif chemokine ligand 12 (CXCL12), which appears to confer heightened resistance to gemcitabine by inducing the autocrine production of IL-6 [25,26].

### 2.2. Tumor-Associated Macrophages (TAMs)

TAMs, a major immune component of the pancreatic adenocarcinoma TME, exhibit pro-tumor functions that promote growth, invasion, and metastasis. TAMs are polarized to an M2 phenotype in response to factors such as macrophage colony-stimulating factor (M-CSF), IL-4, IL-10, and TGF-β secreted by PDAC cells [27]. M2 TAMs suppress anti-tumor immune responses, stimulate angiogenesis and lymphangiogenesis, and secrete factors that support ECM remodeling, cancer cell invasion, and metastasis. Pancreatic tumor cells initiate the secretion of CCL2 shortly after undergoing malignant transformation. C-C motif chemokine ligand 2 (CCL2) acts as a potent chemoattractant for monocytes and macrophages signaling through C-C chemokine receptor type 2 (CCR2) and type 4 (CCR4) and plays a crucial role in recruiting TAMs and promoting tumorigenesis. Within the PDAC TME, immunosuppressive factors, like transforming TGF-β and IL-10, facilitate the differentiation of monocytes into TAMs that produce CCL2 in response, establishing a positive feedback loop. Furthermore, CCL2 expression can also be observed in later stages of tumor growth and formation, contributed by CAFs [28].

To conclude, TAMs play a significant role throughout all stages of cancerous development, shaping an immune-suppressive TME, thus emerging as a promising immunotherapeutic target.

### 2.3. Adipocytes

Adipocytes represent an important component of the PDAC TME that influences tumor progression. It appears that prolonged exposure to cancer cells prompts adipocytes derived from obese individuals to undergo phenotypic transformations. These changes involve the depletion of lipid contents and the acquisition of fibroblast/myofibroblast-like characteristics, thereby contributing to the cellular pool of CAFs [29]. PSCs’ functional genes include fatty acid-binding protein 4 (FABP4), associated with lipid transport and retinoid storage, and adipogenesis regulatory factor (ADIRF), associated with adipogenesis. This genetic signature seems to be enriched in the stellate-like fibroblast TME, which is thought to be the main source of CAFs in PDAC [30]. Factors secreted by adipocytes, such as adipokines, pro-inflammatory cytokines, and fatty acids, promote malignant cell growth, metabolism, invasion, and therapy resistance [29]. Furthermore, neoplastic cells secrete exosomes and factors such as TNF-α that alter adipocyte function, creating a feed-forward loop that fosters tumor progression [31]. Targeting the interactions between neoplastic cells and adipocytes may represent a novel strategy to inhibit tumor growth and overcome treatment resistance.

### 2.4. Neural Cells

There is evidence linking tumor progression with prominent perineural alterations, such as neural hypertrophy, neural density, and neural remodeling. The release of elevated levels of acetylcholine from the vagus nerve in PDAC seems capable of reprogramming the immune tumor environment. Studies have unveiled that perineural invasion triggers an intense cholinergic signaling surge and hinders the recruitment of CD8^+^ T cells inducing the epigenetic suppression of CCL5 in cancer cells. Additionally, it directly inhibits interferon gamma (IFNγ) production, fostering a shift from a T-helper type 1 cell (Th1) to T-helper type 2 cell (Th2) immune response [32].

Additionally, Schwann cells could potentially exert a notable influence on the TME by engaging in interactions with macrophages, mast cells, DCs, myeloid-derived suppressor cells (MDSCs), and diverse immune cell types. These findings underscore the crucial role of perineural invasion in fostering an immunosuppressive microenvironment, thus highlighting it as a promising target for immunotherapy [33].

### 2.5. Endothelial Cells and Pericytes

In PDAC, the TME showcases an abnormal pericyte phenotype characterized by elevated αSMA expression, influenced by exosomes derived from cancer cells. This distinctive αSMA^+^ pericyte subset displays modified biomechanical attributes along with an immunomodulatory phenotype, possibly fostering the hypoxic and immunosuppressive milieu within the TME [34]. PDAC affects endothelial cells, leading to reduced platelet endothelial cell adhesion molecule 1 (PECAM1) expression and increased P-selectin and intercellular adhesion molecule 1 (ICAM1) expression. Additionally, pericytes demonstrate an elevated expression of CD274, P-selectin, and E-selectin, suggesting a potential role in intercepting extravasating T cells and hindering their activation and proliferation [34].

## 3. Emerging Therapeutic Strategies

The TME is characterized by a complex network of signaling pathways that contribute to tumor initiation, growth, invasion, metastasis, and drug resistance. Understanding the complex interplay between cellular components and innovative pathways in the PDAC TME is crucial for the development of effective therapeutic strategies. Targeting the TME has emerged as a promising approach to improve treatment outcomes. Strategies aimed at inhibiting CAF activation, modulating the immune response, targeting angiogenesis, and disrupting neural cell interactions are being actively investigated. Several preclinical models and ongoing clinical trials are evaluating the efficacy of these strategies, holding promise for the future management of PDAC [35,36].

Furthermore, the occurrence of bone metastasis as the primary presentation highlights the need for early detection and tailored interventions. Moreover, understanding the molecular characteristics and clinical aspects of skeletal metastases is essential for effective management. These findings underscore the complexity of pancreatic malignancy and the importance of multidisciplinary approaches. Further research is needed to validate these discoveries and explore additional molecular alterations driving disease progression and treatment resistance [37].

### 3.1. Targeting Signaling Pathways

Recent studies have advanced our understanding of pancreatic cancer biology, TME, and treatment response. The identification of dysregulated pathways, lifestyle factors, and novel biomarkers offers potential for targeted therapies and personalized medicine [18]. Clinical trials targeting different dysregulated pathways are already ongoing and summarized in Table 1.

Oncogenic KRAS mutations are found in over 90% of PDAC cases and drive tumor initiation, progression, and maintenance [7]. Activated KRAS signaling promotes cell proliferation, survival, invasion, metastasis, and stemness through effectors that are well-known genetic drivers, such as rapidly accelerated fibrosarcoma kinase (RAF)–dual specificity mitogen-activated protein kinase (MEK)–extracellular signal-regulated kinases (ERK), phosphatidylinositol 3-kinases (PI3K)–protein kinase B (AKT), and ral guanine nucleotide dissociation stimulator (RAL-GDS) [44,45,46]. Direct targeting of oncogenic KRAS has been challenging, necessitating alternative approaches a.k.a. targeting downstream effectors or synthetic lethal interactions [46]. For example, inhibitors of the RAF–MEK–ERK and PI3K–AKT pathways have been developed and are currently being tested in clinical trials. In addition, synthetic lethality approaches that target vulnerabilities in cells with oncogenic KRAS mutations have shown promise in preclinical models [47].

In dysregulated signaling pathways, such as TGF-β, Notch, and PD-L1, stromal-derived factor-1 (SDF-1)/CXCR4 signaling pathways play key roles in pancreatic cancer progression and treatment resistance. Lifestyle factors, including alcohol consumption, have been linked to altered TGF-β signaling in pancreatic cancer patients. The TME, particularly CAFs and their interaction with mast cells, have been shown to activate TGF-β signaling and confer resistance to specific therapies [48]. Additionally, literature data have outlined a distinctive biological profile in pancreatic sarcomatoid carcinoma, a rare pancreatic histotype [49]. These findings lay the foundation for future investigations into the potential interaction between the PD-L1/programmed cell death protein 1 (PD-1) axis and Notch pathways, thus catalyzing the development of innovative therapeutic strategies [50].

The pro-tumor role of HIF-2α in PDAC has been demonstrated both in vivo and in vitro, as it promotes tumor proliferation, invasion, stemness, and angiogenesis. Notably, HIF-2α interacts with β-catenins, leading to increased activity of the classical Wnt/β-catenin pathway. Furthermore, the formation of the HIF-2α/β-catenin complex stabilizes HIF-2α and enhances its transcriptional activity. Importantly, patients with high levels of HIF-2α expression have a poor prognosis, indicating that HIF-2α is a promising therapeutic target [51].

Building upon the results, the WNT pathway emerges as a critical nexus, bridging the biological hallmarks of invasive disease with the permissive immune environment. Through in vitro experiments, the effectiveness of WNT pathway targeting by use of the potent Tankyrase inhibitor XAV939 has been demonstrated. Notably, treatment with XAV939, which antagonizes WNT signaling via the stimulation of β-catenin degradation, not only impedes tumor cell migration but also enhances the immune response against cancer cells, presenting an encouraging theragnostic avenue for patients afflicted with nodal-positive PDAC [52].

Furthermore, accumulating evidence from several studies underscores the significance of angiopoietin-like 4 (ANGPTL4) and macrophage stimulating 1 (MST1) as pivotal regulators of invasiveness, with profound prognostic implications. These findings lay the groundwork for robustly powered clinical investigations, poised to advance our understanding and management of PDAC [53,54].

### 3.2. Chemokines in the PDAC Tumor Microenvironment

Chemokines play a crucial role in pancreatic carcinoma, impacting numerous aspects of tumor progression and immune response. They are essential for orchestrating the migration and recruitment of immune cells within the tumor microenvironment (TME). By facilitating the recruitment of immunosuppressive cells such as myeloid-derived suppressor cells (MDSCs), regulatory T cells, TAMs, and tumor-associated neutrophils (TANs), chemokines contribute to the creation of an immunosuppressive TME. This, in turn, inhibits the anti-tumor immune responses executed by CD4+/CD8+ T lymphocytes and NK cells [55].

Certain chemokines, such as CXCL12 and its receptor CXCR4, promote tumor cell migration and invasion, facilitating metastasis to distant organs. CXCR4, activated by its ligand CXCL12 (also known as SDF-1), plays a crucial role in promoting the migration and invasion of tumor cells in pancreatic ductal adenocarcinoma (PDAC). This receptor initiates intracellular signaling pathways such as Akt, ERK, and NF-κB, which are essential for regulating cell survival, proliferation, and motility, thereby facilitating tumor progression and metastasis. CXCR4 not only enhances tumor cell adhesion to extracellular matrix components and endothelial cells but also promotes their chemotactic movement through tissues, facilitating invasion into blood vessels and distant organs [56,57].

The expression levels of specific chemokines and their receptors can serve as predictive and prognostic biomarkers [58,59].

They may also represent targets for new therapeutic and tumor imaging strategies. For instance, alterations in chemokine profiles could be used to predict the radiosensitivity of PDAC tumors and help stratify patients for radiotherapy [60]. Modulating chemokine pathways through specific inhibitors, antibodies, or other therapeutic strategies could improve the efficacy of current treatments, bolster the immune response against the tumor, and constrain disease progression.

### 3.3. Checkpoint Inhibitors in Clinical Trials

Recent therapeutic strategies for the PDAC treatment are increasingly embracing a multifaceted approach, capitalizing on a combined modality that targets various facets of the immune TME. By engaging crucial checkpoints within pathways pivotal for tumor progression, these emerging therapeutic strategies aim to disrupt the intricate web of tumor–immune interactions, potentially offering more effective outcomes in the management of PDAC. For instance, single-agent ipilimumab and tremelimumab exhibited ineffectiveness in treating advanced pancreatic cancer [61]. Conversely, combination therapy with ipilimumab and gemcitabine has shown promise by enhancing the immune response through increased naïve T-cell activation [61].

Furthermore, a high tumor mutational burden (TMB) in cancer cells often results in the production of more immunogenic neoantigens and may function as a predictor of response to immunotherapy. An ongoing phase II clinical trial (NCT05093231) is examining the efficacy of pembrolizumab in combination with Olaparib in patients with metastatic pancreatic adenocarcinoma who have a high tumor mutation burden, with results anticipated in 2026 [62].

Based on insights gleaned from ongoing clinical trials, forthcoming research should prioritize integrated approaches that combine immune checkpoint inhibitors (ICIs) with diverse therapeutic modalities, encompassing chemotherapy, radiotherapy, and novel immunotherapy platforms like cancer vaccines and adoptive cell transfer. This multifaceted approach holds promise for enhancing treatment outcomes in pancreatic cancer [63].

### 3.4. PDAC Resistance to ICI: Unraveling the Mechanisms

ICIs, such as those targeting PD-1/PD-L1 and CTLA-4, have revolutionized cancer therapy by enhancing the immune system’s ability to recognize and destroy cancer cells. Despite their success in various cancers, PDAC has shown limited responsiveness to ICIs due to several intrinsic and extrinsic factors.

Immunosuppressive Tumor Microenvironment: PDAC is characterized by a highly immunosuppressive microenvironment. This microenvironment is populated by Tregs, MDSCs, TAMs that collectively inhibit effective anti-tumor immune responses. These cells secrete cytokines and growth factors that suppress cytotoxic T-cell activity and promote tumor growth [64].

- Desmoplastic Stroma: The dense stromal matrix in PDAC acts as a physical barrier to immune cell infiltration. The stromal cells, primarily pancreatic stellate cells, produce extracellular matrix components that not only impede T-cell access to the tumor but also support the survival and proliferation of immunosuppressive cells. This desmoplastic reaction further contributes to the immunosuppressive microenvironment [65].
- Low Tumor Mutational Burden (TMB): PDAC typically has a lower TMB compared with other cancers such as melanoma and non-small cell lung cancer. A lower TMB results in fewer neoantigens being presented on the tumor cell surface, which reduces the immunogenicity of the tumor. Consequently, there are fewer targets for the immune system to recognize and mount an effective response [66].
- Low PD-L1 Expression: PDAC tumors often exhibit low levels of PD-L1 expression. PD-L1 is the ligand for PD-1, and its expression on tumor cells is a key mechanism by which tumors evade immune surveillance. The efficacy of ICIs, particularly those targeting the PD-1/PD-L1 axis, is diminished in tumors with low PD-L1 expression due to the reduced interaction between PD-1 on T cells and PD-L1 on tumor cells [67].

Given the challenges in treating PDAC with ICIs alone, current research is focusing on combination therapies that can enhance the effectiveness of ICIs by modifying the tumor microenvironment and increasing the immunogenicity of the tumor. Combining ICIs with chemotherapy can induce immunogenic cell death, enhancing tumor antigen presentation and stimulating an anti-tumor immune response. For example, gemcitabine and nab-paclitaxel are commonly used in PDAC and have been shown to modulate the immune environment [68]. Combination with targeted therapies can also be effective. MEK inhibitors, for instance, alter tumor signaling pathways involved in immune evasion, modulate the tumor microenvironment, and enhance the efficacy of ICIs by reducing MDSCs and Tregs [69].

In Table 2 below, we provide an overview of ongoing and completed clinical trials investigating various combination strategies to overcome resistance to ICIs in PDAC.

### 3.5. Cancer Cachexia Mechanisms

Cachexia is described in 90% of all patients with PDAC and is associated with reduced survival and higher rates of metastatic disease. It is characterized by pathological weight loss due to excessive wasting of skeletal muscle and adipose tissue mass, even though there is still a lack of consensus on its definition, which complicates the standardization of diagnosis and treatment. Its pathogenetic mechanisms are still to be uncovered, but our understanding of interested genes, proteins, and cytokines has evolved considerably. Tumor invasion is associated with digestive disorders and malabsorption because of loss of pancreatic function, eventually resulting in cachexia. The inflammatory response in the liver plays a crucial role characterized by the production of inflammatory compounds and mediators, such as C-reactive protein (CRP), IL-6, IL-1β, IL-8, and TNF-α. This inflammatory milieu underscores its significant impact on hepatic function and systemic inflammation in various disease states [74].

Systemic inflammation in patients with PDAC could stimulate cytokine-mediated Atrogin-1/MAFbx expression that participates in the process. Emerging evidence indicates that epigenetic factors regulate both muscle development and cancer cachexia pathways [75]. Noncoding RNAs (ncRNAs) have specifically been implicated in influencing the initiation and progression of cachexia, with potential applications as biomarkers for its detection [76]. Despite extensive research identifying numerous drug targets in animal models, very few pharmacological treatments have successfully transitioned into clinical practice, and no monotherapy has proven effective. Therefore, a multimodal treatment approach is widely recognized as essential. This approach includes nutritional support and exercise to stabilize weight, alongside pharmacological interventions targeting inflammatory and metabolic changes. Additionally, addressing secondary symptoms that exacerbate cachexia, such as loss of appetite, gastrointestinal tract impairment, chronic pain, fatigue, and depression, is crucial [77].

Furthermore, systemic metabolic disturbances mediated by pro-cachectic factors, systemic inflammation, and epigenetic alterations underscore that PDAC is a systemic disease rather than a localized organ defect, affecting multiple organs beyond the pancreas. The endocrine organ-like tumor hypothesis emphasizes the systemic effects of PDAC leading to tissue wasting [78]. Consequently TGF-β, a known inducer of Krueppel-like factor (KLF10) and cachexia-promoting factors, has been identified by bioinformatic analyses as a key upstream regulator of KLF10. Lately, the strategy of mimicking KLF10 loss of function has been proposed as a potential approach to alleviate cancer-associated muscle wasting [79].

### 3.6. Gene Mutation in PDAC

The KRAS gene mutation has been found in 90% of pancreatic cancer patients. Mutant KRAS can trigger a series of well-differentiated precursor lesions, including pancreatic intraepithelial neoplasia. Mutations of KRAS involve codons G12, G13, and Q61, while recurrent mutations in K117 and A146 appear to be additional hotspots [80].

Mutations in the TP53 gene are the second most common in PDAC [81]. TP53 dysfunction disrupts the immune landscape within the microenvironment, fostering an inflammatory milieu that drives tumor development. Consequently, the inactivation of wild-type TP53 activity directly affects cell cycle progression, apoptosis, and senescence [13].

Mutations in CDKN2A are present in up to 95% of cases, with a familial genetic predisposition observed. Hypermethylation of the CDKN2A promoter is a confirmed marker of CDKN2A inactivation, playing a significant role in malignant tumor development. In the context of PDAC, transcriptional silencing mechanisms, particularly involving CDKN2A, emerge as significant targets for therapeutic intervention. Furthermore, insights gleaned from promoter methylation offer valuable perspectives for enhancing the diagnosis and treatment strategies for patients [82].

Studies revealed that the absence of SMAD4 correlated with diminished lymphocyte infiltration, decreased expression of T-cell markers, and attenuated T-cell-mediated cytotoxicity [83]. Patients with intact SMAD4 experience enhanced survival, while SMAD4 loss leads to impaired synthesis of immune-related molecules, compromised T-cell activation, and decreased PD-L1 expression. Understanding these genetic mutations is crucial for developing targeted therapies and improving the clinical management of PDAC [83].

### 3.7. K-Ras Inhibitors: Revolutionizing Pancreatic Cancer Treatment

K-Ras inhibitors represent a significant advancement in the treatment of pancreatic cancer, which is notoriously challenging to treat due to its aggressive nature and late-stage diagnosis. The K-Ras protein, encoded by the KRAS gene, is a critical player in cell signaling pathways that regulate cell growth, differentiation, and survival.

Mutations in KRAS are found in over 90% of pancreatic ductal adenocarcinoma (PDAC) cases, making it a prime target for therapeutic intervention [84]. For decades, targeting K-Ras has been difficult due to the protein’s structure and the complexity of its interactions within the cell. The K-Ras protein lacks deep binding pockets, making it challenging for small molecules to effectively inhibit its function. However, recent breakthroughs have led to the development of specific K-Ras inhibitors, such as sotorasib (AMG 510), which can effectively target the mutant form of the protein. These inhibitors work by binding to the mutant K-Ras protein, specifically the G12C mutant, thereby blocking its activity and disrupting the downstream signaling pathways that drive tumor growth and proliferation [85]. Clinical trials have demonstrated promising results with K-Ras inhibitors. For instance, sotorasib has shown improved response rates and extended progression-free survival in patients with KRAS(G12C)-mutant pancreatic cancer. In a phase I clinical trial, sotorasib achieved a disease control rate of 88% among heavily pretreated patients, with a median progression-free survival of 6.9 months [86].

These drugs have shown the ability to shrink tumors and, in some cases, halt disease progression, offering new hope for patients who previously had limited treatment options [87]. The success of K-Ras inhibitors also opens the door for combination therapies, where these inhibitors can be used alongside other treatments such as chemotherapy, immunotherapy, or other targeted agents to enhance their efficacy. This multimodal approach aims to overcome drug resistance and achieve more durable responses. For example, combination strategies with immune checkpoint inhibitors are being explored to potentiate the anti-tumor immune response and improve clinical outcomes [88]. Overall, the advent of K-Ras inhibitors marks a paradigm shift in the treatment of pancreatic cancer, providing a targeted and effective strategy against one of the most challenging oncogenic drivers. Continued research and clinical development will be essential to fully realize the potential of K-Ras inhibitors and to improve outcomes for patients with pancreatic cancer. This includes exploring inhibitors for other KRAS mutations and developing strategies to address potential resistance mechanisms [89].

## 4. Future Perspectives

In a recent study by Zhang et al. [90], researchers used a comprehensive multi-omic strategy to pinpoint a set of signature genes linked to anoikis in pancreatic cancer. They analyzed data from extensive cancer genomic databases, such as the Cancer Genome Atlas (TCGA) and the Gene Expression Omnibus (GEO), along with single-cell sequencing datasets and in vitro assays, to thoroughly investigate the molecular mechanisms underlying anoikis resistance [90].

By integrating diverse data sources and experimental approaches, researchers have identified a panel of characteristic genes with potential roles as prognostic biomarkers in pancreatic cancer. These genes could offer the opportunity to predict patient outcomes, enabling clinicians to accordingly adapt treatment strategies. Moreover, the identified signature genes may also help to predict the sensitivity of pancreatic cancer cells to chemotherapy, aiding in the selection of appropriate chemotherapy regimens. The findings of Zhang et al. [90] contribute to the growing body of knowledge surrounding pancreatic cancer and its resistance mechanisms. The identification of signature genes associated with anoikis resistance opens new avenues for personalized medicine and targeted therapies [90].

However, further validation and functional characterization of these genes are necessary to fully exploit their clinical potential. It is crucial to emphasize the importance of integrating multiple data sources and experimental approaches in cancer research. By combining genomics, transcriptomics, proteomics, and functional assays, researchers can obtain a more comprehensive and nuanced understanding of the complex biological processes underlying cancer progression and treatment resistance. This integrated approach facilitates the discovery of new biomarkers and therapeutic targets, ultimately leading to improved patient outcomes and more effective cancer treatments.

Despite significant efforts in scientific research, PDAC remains one of the greatest therapeutic challenges. To date, only modest progress has been made in the diagnosis and management of the disease. The TME of PDAC is characterized by an intense desmoplastic reaction, hypoxia, acidosis, high interstitial pressure, and considerable heterogeneity [90]. The complex crosstalk between malignant and stellate cells underpins the desmoplastic reaction, which is closely associated with cancer invasion, progression, and metastasis [90].

Furthermore, the combination of desmoplasia and high interstitial pressure obstructs the poorly vascularized tumor, exacerbating hypoxia. These factors collectively form a mechanical barrier that hinders the access of chemotherapeutic drugs [91].

Significant progress has been made due to discoveries over the past decade. Research has shown that extracellular acidosis creates a favorable environment for tumor cells, forming a chemical barrier that impedes immune surveillance and diminishes the effectiveness of chemotherapy [92].

PDAC demonstrates resilience in a nutrient-deregulated environment, where dysregulated metabolic pathways and altered nutrient availability contribute to its progression and survival. For instance, it relies on elevated levels of autophagy and other lysosomal processes [93]. Targeting metabolic pathways involving glucose or lipid metabolism, autophagy, or ROS production represent future possibilities yet to be explored. Recent studies using both in vitro experiments and animal models have demonstrated that SIRT4 can inhibit tumor growth and enhance autophagy in PDAC through mechanisms involving the suppression of glutamine metabolism and the consequent phosphorylation of the p53 protein. Moreover, the activation of AMPK and PPAR-γ and the inhibition of GSK-3β were demonstrated to significantly block tumor growth in pancreatic cancer and increase the sensitivity to conventional chemotherapies, such as gemcitabine and TMZ. Moreover, cachexia can also be targeted simultaneously by the same drugs since they could block inflammatory and NF-κB-dependent cascades [94].

It is known that tumor cells use glutamine (Gln) to support proliferation and redox balance. Pilot studies suggested that broadly targeting Gln metabolism could provide a therapeutic avenue and that combination with an ERK signaling pathway inhibitor could act synergistically [95]. The metabolic intermediates of the tricarboxylic (TCA) cycle play a significant role in the metabolic aberrations in PDAC. Recent research suggests that enzymes regulating metabolic pathways, including glycolysis and the TCA cycle, play pivotal roles in metabolic alterations and could serve as novel therapeutic targets [96]. Very common genetic mutations observed in PDAC involve alterations in KRAS and p53 proteins, which play significant roles in regulating ROS production and control, respectively. Combining the promotion of ROS production with the inhibition of antioxidant capacity shows promise as a therapeutic strategy [97].

Moreover, an in vivo genetic screen identified multiple glycolysis genes as potential targets to enhance tumor cell sensitivity to MEK inhibition. Molecular and metabolic analyses revealed that co-targeting glycolysis and MAPK signaling induces apoptosis through endoplasmic reticulum stress, suggesting that this combination could effectively target KRAS-driven PDAC [98].

Primarily stemming from dysregulated cancer metabolism, this acidic environment is largely induced by the secretion of metabolic end products and cytokines. These substances prompt interactions between cancer cells and stromal cells, fostering tumor progression and aggressiveness. Notably, the acidic microenvironment serves as a catalyst for the metastatic cascade, activating processes such as epithelial-mesenchymal transition (EMT), ECM degradation, and tumor cell migration [99].

The intricate TME, encompassing mechanisms such as the desmoplastic reaction, hypoxia, limited vascular access, acidic extracellular pH, and the production of drug extruder proteins, whether acting in concert or independently, contributes to the persistent challenge of low chemosensitivity to many chemotherapeutic agents. This complexity underscores the significance of TME components in immune system suppression and tumor progression, a feature partly distinct from other solid and hematological malignancies [100,101,102,103,104,105].

Given that PDAC drug resistance is rooted in this unique hostile microenvironment and metabolic reprogramming, it presents an opportunity for potentially innovative strategies in the future treatment of patients [92].

Hence, the targeting of desmoplasia and/or cancer metabolism, either alone or in combination with other targeted agents or cytotoxic compounds, holds promise as therapeutic strategies that could prove beneficial for improving patient outcomes and extending the survival rate [106].

## 5. Conclusions

Collectively, PDAC is a devastating disease characterized by a hostile TME that promotes tumor growth and metastasis. Emerging evidence underscores the significance of comprehending the multifaceted roles played by the intricate components of the TME in both tumor suppression and progression.

Future therapeutic approaches should prioritize targets to holistically reprogram the TME rather than solely focusing on depleting specific components. Combination or multimodal strategies targeting multiple aspects of the TME simultaneously hold promise for success. Such approaches must carefully consider the complementarity of the targeted pathways. When devising novel therapeutic strategies, it is crucial to explore whether certain TME features are organ specific and should be taken into consideration when treating metastatic cancers. Moreover, the potential feedback responses to treatments must be carefully considered and leveraged, considering the effects of preceding conventional therapeutic interventions.

As technology advances, multiplexed imaging, immunophenotyping, and mutational analysis tools become increasingly high throughput, and the goal of personalized treatment tailored to the TME characteristics of individual patients is steadily approaching reality.

Despite setbacks in many trials, the growing understanding of the PDAC microenvironment and the emergence of innovative strategies provide reasons for optimism regarding future successful treatment.

## Figures and Tables

**Table 1:** Overview of innovative therapeutic strategies targeting known dysregulated pathways in PDAC currently undergoing advanced clinical trials.

Molecular Target	Mechanism of Action	Promising Agents	Combination Partner	Study Phase	Reference
MEK	Multiple pathway inhibition	Trametinib	ABT-263 (Navitoclax, BCL-XL inhibitor)	Xenografts	[37]
	MEK inhibitors as backbone	Selumetinib	BKM120 (Buparlisib, PI3K inhibitor)	Mouse model	[38]
	Synthetic lethality	Trametinib	SHP099 (SHP2 inhibitor)	Mouse model	[38]
			SHOC2 knockout		[38]
	Immunosuppressive TME modulation	Cobimetinib	CD40 antibody	Mouse model	[38]
		Trametinib	Palbociclib and PD-L1 antibody	Mouse model	[38]
TGF-β	Block TGF-β receptor reducing tumor growth and immunosuppression	Galunisertib	Gemcitabine	Phase I/II	[39]
Notch	Inhibit γ-secretase reducing tumor proliferation and invasion	Nirogacesta	Gemcitabine	Phase I/II	[40]
HIF-1α	Reduce angiogenesis, increase sensitivity to chemotherapy	FOLFIRINOX *	Digoxin	Phase II	[41]
Wnt	Prevent the interaction between Wnt proteins and membrane-bound Frizzled receptors	Vantictumab	Nab-Paclitaxel and Gemcitabine	Phase I	[42]
		Ipafricept	Nab-Paclitaxel and Gemcitabine	Phase I	[43]

* FOLFIRINOX = regimen comprising folinic acid, fluorouracil, irinotecan, and oxaliplatin.

**Table 2:** Strategies to overcome resistance to ICIs in PDAC.

Clinical Trial	ICI	Combination Drug(s)	Phase	Status	Description
NCT04104672	Nivolumab (PD-1)	Chemotherapy (Gemcitabine, Nab-Paclitaxel)	Phase II	Recruiting	Evaluates the efficacy and safety of nivolumab combined with chemotherapy [70]
NCT04548752	Pembrolizumab (PD-1)	Olaparib (PARP inhibitor)	Phase II	Recruiting	Testing the addition of pembrolizumab, an immunotherapy cancer drug, to olaparib as therapy for patients with pancreatic cancer that has spread with inherited BRCA mutations [71]
NCT02558894	Durvalumab (PD-L1)	Tremelimumab (CTLA-4)	Phase II	Active, not recruiting	Studies the combination of durvalumab and tremelimumab in PDAC [72]
NCT03193190	Atezolizumab (PD-L1)	Cobimetinib (MEK inhibitor)	Phase I/II	Completed	Explores the combination of atezolizumab and cobimetinib in advanced PDAC [73]

## Data Availability

No new data were created or analyzed in this study. Data sharing is not applicable to this article.
